# The Full-Length Transcriptome Provides New Insights Into the Transcript Complexity of Abdominal Adipose and Subcutaneous Adipose in Pekin Ducks

**DOI:** 10.3389/fphys.2021.767739

**Published:** 2021-11-10

**Authors:** Dandan Sun, Xiaoqin Li, Zhongtao Yin, Zhuocheng Hou

**Affiliations:** Department of Animal Genetics, Breeding and Reproduction, College of Animal Science and Technology, China Agricultural University, Beijing, China

**Keywords:** Pekin duck, abdominal adipose, subcutaneous adipose, full-length transcriptome, alternative splicing, proliferation, differentiation

## Abstract

Adipose tissues have a central role in organisms, and adipose content is a crucial economic trait of poultry. Pekin duck is an ideal model to study the mechanism of abdominal and subcutaneous adipose deposition for its high ability of adipose synthesis and deposition. Alternative splicing contributes to functional diversity in abdominal and subcutaneous adipose. However, there has been no systematic analysis of the dynamics of differential alternative splicing of abdominal and subcutaneous adipose in Pekin duck. In our study, the Pacific Biosciences (PacBio) Iso-Seq technology was applied to explore the transcriptional complexity of abdominal and subcutaneous adipose in Pekin ducks. In total, 143,931 and 111,337 full-length non-chimeric transcriptome sequences of abdominal and subcutaneous adipocytes were obtained from 41.78 GB raw data, respectively. These data led us to identify 19,212 long non-coding RNAs (lncRNAs) and 74,571 alternative splicing events. In addition, combined with the next-generation sequencing technology, we correlated the structure and function annotation with the differential expression profiles of abdominal and subcutaneous adipose transcripts. This study identified lots of novel alternative splicing events and major transcripts of transcription factors related to adipose synthesis. STAT3 was reported as a vital gene for adipogenesis, and we found that its major transcript is STAT3-1, which may play a considerable role in the process of adipose synthesis in Pekin duck. This study greatly increases our understanding of the gene models, genome annotations, genome structures, and the complexity and diversity of abdominal and subcutaneous adipose in Pekin duck. These data provide insights into the regulation of alternative splicing events, which form an essential part of transcript diversity during adipogenesis in poultry. The results of this study provide an invaluable resource for studying alternative splicing and tissue-specific expression.

## Introduction

Duck is one of the most widely distributed waterfowl in the world. After being artificially domesticated, Pekin duck stores a large amount of lipids in abdominal and subcutaneous adipose tissues (Kou et al., 2012; Lin et al., 2018), which is an ideal animal model for studying the fat deposition process of birds. The previous research showed that the lipid deposition patterns of the two tissues were different during the growth periods, the content of subcutaneous adipose tissue was higher than abdominal adipose tissue (Ding et al., 2012). However, the difference of molecular mechanism of lipid deposition with different adipose tissues of Pekin duck is still not clear.

Accompanied by the progress of sequencing technology, single-molecule sequencing was widely used in plant research, such as corn (Guo et al., 2021), rice (Du H. et al., 2017), clover (Chao et al., 2018), bamboo (Wang et al., 2017), etc. highlighting the huge advantages of identification of alternative splicing by full-length transcriptome. Single-molecule sequencing can directly obtain all information of the RNA sequence without assembly. However, there are fewer applications of single-molecule sequencing technology in bird research. For ducks, only PacBio Sequel was used to sequence the full-length transcriptome of eight Pekin duck tissues, and identified 35,031 alternative splicing events among 3,346 genes (Yin et al., 2019).

Fortunately, the assembly of the high-quality Mallard genome provides a vital support for us to accurately identify the isoforms and alternative splicing events of genes (NCBI, 2019). Alignment of different isoforms to the reference genome can effectively identify the modes of alternative splicing of genes, especially to improve the accuracy of long isoforms alignment (Kraft and Kurth, 2020). At present, the process of alternative splicing identification for species with reference genome is mature and reliable (Song et al., 2020; Xu et al., 2021). Combined analysis using transcriptome of short-read and long-read sequencing can further improve gene structure annotation, verify splicing sites, analyze tissue-specific or time-specific expression of different isoforms (Hu et al., 2021).

Another advantage of single-molecule transcriptome sequencing is the identification of TFs (Mazzocca et al., 2021) and lncRNAs (Troskie et al., 2021) in tissues, which is essential for the study of transcriptional regulation of fat-related biological processes. Adipogenesis is a process characterized by a complex network involving many TFs and lncRNAs that regulate gene expression (Squillaro et al., 2020). LncRNAs process multiple cellular functions and regulate chromatin remodeling, transcriptional and post-transcriptional events to affect gene expression. Recent investigations have shown that these molecules play a key role in regulating the development and activity of the white and brown/beige adipogenic process (Squillaro et al., 2020). Overexpression and knockout methods have been widely used to understand the contribution of TFs to adipocyte development, providing a basic strategy for studying the complexity of adipogenesis *in vitro*. So far, more than 12 transcription factors have been shown to play an important role in adipocyte development (Farmer, 2006; Lee et al., 2019; Ambele et al., 2020; Zhang et al., 2020). Comprehensive analysis of different isoforms of TFs during adipocyte multiple developmental time points can broaden our view of regulation of different adipocyte development in birds.

Therefore, we performed ISO-seq and RNA-Seq analysis on abdominal and subcutaneous adipocytes derived from the Pekin ducks. We addressed the proliferation and differentiation of the abdominal and subcutaneous adipocytes to identify potential differences in isoforms. The results of this analysis allowed us to expand our cognition of alternative splicing and differential expression which indicate different regulation modes, and provide a rich resource into the alternative splicing that forms an essential part of transcript diversity and complexity during abdominal and subcutaneous adipose synthesis and deposition. These results will facilitate future functional genomics studies and broaden our horizons of alternative splicing in poultry.

## Materials and Methods

### Cell Culture and Differentiation Induction

The cell samples used in this experiment were primary preadipocytes isolated from the abdominal and subcutaneous adipose tissues of Pekin ducks provided by Beijing Golden Star Ltd. The experimental procedure was in accordance with the guidelines of the China agricultural University Animal Care Committee. The isolation method referred to the method used in our previous study (Wang et al., 2019). Ducks were sacrificed under deep anesthesia with sodium pentobarbital (Sigma). Abdominal and subcutaneous adipose tissue was collected under sterile conditions and washed with PBS. The clean adipose tissue was minced into fine sections and digested with 15 mL of digestion Solution [DMEM/F12 (Dulbecco’s modified Eagle’s medium/Ham’s nutrient mixture F-12), 100 mM HEPES, 4% BSA, 2 mg/mL collagenase I (Invitrogen), pH 7. 4] for 65 min at 37°C in a water bath shaker. After incubation and stop digestion by growth medium (DMEM/F12, 10% FBS, 100 U/mL penicillin, and streptomycin). The mixture was filtered through nylon screens with 70 μm mesh openings to remove undigested tissue and large cell aggregates. The filtered suspensions were centrifuged at 300 × *g* for 10 min to separate floating adipocytes from preadipocytes. The harvested preadipocytes were then re-suspended with 10 mL of Blood Cell Lysis Buffer (Invitrogen), and incubated at room temperature for 10 min. The abdominal and subcutaneous preadipocytes isolated were inoculated in a growth medium. The cell culture was carried out at 5% CO_2_ concentration, 37°C and 95% air humidity. Preadipocytes can be induced to differentiate by adding oleic acid to the growth medium (Shang et al., 2014).

### Cell Counting Kit-8 Assay

Cell Counting Kit-8 (CCK-8) is a highly sensitive colorimetric assay for cell proliferation. In order to determine the difference of proliferation rate of preadipocytes in different parts of Pekin duck, abdominal and subcutaneous preadipocytes divided into 4 × 10^3^ cells/well were seeded in 96 wells cell culture plate. 100 ul medium was added to each well. After induction for 24 h, 48 h, 96 h, 144 h, 192 h, and 240 h, 10 ul CCK-8 (Dojindo Laboratories, JP) was added to the sample well, incubated at 37°C for 2 h, and the absorbance value at 450 nm was measured by the multi-function microplate reader (Infinite F200, CH).

### Determination of the Activity of Glycerol-3-Phosphate Dehydrogenase

Glycerol-3-phosphate dehydrogenase (GPDH) is a rate-limiting enzyme of fatty acyl-CoA biosynthesis, and its enzyme activity increases significantly in the late stage of adipose differentiation, so it can be an index to characterize the differentiation degree of preadipocytes. In our experiment, abdominal and subcutaneous preadipocytes were selected at 0 h, 48 h, and 96 h after inducing differentiation. GPDH activity was conducted by using GPDH activity detection kit (Sigma, United States). Three biological replicates (*n* = 3) were included at each time point. Bovine serum albumin was used as the standard, BCA protein detection kit (Sigma, United States) was used to determine the protein concentration of cell culture homogenate (Matsubara et al., 2005).

### Determination of Relative Lipid Droplet Content

Oil red O (Sigma, United States) staining could specifically stain the neutral lipid in cells because it can be highly dissolved in lipid. In our study, abdominal and subcutaneous preadipocytes were collected at 0 h, 24 h, 48 h, 72 h, 120 h, and 240 h after induction. Firstly, the cells were washed with PBS for three times, fixed with 10% (v/v) paraformaldehyde at room temperature for 30 min, then washed with PBS, stained with 1% oil red O for 40 min, removed the supernatant, and added 1 mL of 100% (v/v) isopropanol to obtain the extraction. The absorbance of the extraction at 500 nm was measured by the multi-function microplate reader (Infinite F200, CH) to characterize the relative lipid droplet content of each sample (Ramirezzacarias et al., 1992). Three biological replicates (*n* = 3) were included at each time point. The data were analyzed by independent sample students’ test.

### Sample Collection and RNA Preparation

All the Pekin duck used in this study were provided by Beijing Golden Star Ltd. Inc. The abdominal and subcutaneous adipose tissues were collected for the primary culture of the preadipocytes. The detailed method of primary cell culture is described in previous research (Matsubara et al., 2005). We collected −48 h (48 h before the initiation of differentiation), 0 h (the initiation of differentiation), 12 h, 24 h, 48 h, and 72 h of abdominal and subcutaneous preadipocytes for RNA extraction respectively. The cleaned adipocytes at all time points were homogenized separately (10 μg per sample) in TRIzol (Invitrogen, United States) and processed according to the manufacturer’s protocol. RNA integrity number (RIN) values were calculated using an Agilent 2100 Bioanalyzer (Agilent Technologies, United States), and RNA concentration was assessed using a NanoDrop^TM^ spectrophotometer (Thermo Fisher Scientific, United States). All RNA samples had an RNA integrity number value >8.0, and an optical density 260:280 ratio >1.9. RNA was then used for mRNA-seq using the Illumina sequencing platform.

### Library Preparation and Pacific Biosciences Sequencing

Abdominal and subcutaneous preadipocytes at 72 h after differentiation were used for full-length transcriptome sequencing. −48 h, 0 h, 12 h, 24 h, 48 h, and 72 h of abdominal adipocytes were collected for RNA-Seq, and each time point included six biological replicates (*n* = 6).

We constructed two Iso-seq libraries for abdominal and subcutaneous adipocytes, which mixed equal amounts of RNA from each sample (5 μg per sample). The libraries were generated according to PacBio Iso-seq sequencing protocol. Briefly, qualified RNA was first obtained for sequence library construction, and the Clontech SMARTer cDNA synthesis kit with Oligo-dT primers was used to generate first-strand and second-strand cDNA from polyA mRNA. Size fractionation and selection (<4 kb and >4 kb) were performed using the BluePippin^TM^ Size Selection System (Sage Science, Beverly, MA, United States). The full-length cDNA was repaired to construct the equal-mole hybrid library. The sequences without joints at both ends of the cDNA were removed. Two SMRT bell libraries were constructed with the Pacific Biosciences DNA Template Prep Kit 2.0 and SMRT sequencing was then performed using the Pacific Bioscience Sequel System. Approximately 5 μg of total RNA was used for mRNA-seq using the Illumina sequencing platform. Suitably sized fragments were selected using AMPure XP beads (Beckman Coulter, United States) to construct the cDNA libraries by PCR. Following construction, double-stranded cDNA libraries were sequenced on an Illumina HiSeq X-10 with PE150 mode (Novogene, CA, United States). The methods of library construction and sequencing were as described elsewhere (Wang et al., 2019). All sequencing data were deposited in National Centre for Biotechnology Information (NCBI) under the BioProject ID PRJNA723918. RNA-Seq data of multiple differentiation stages of subcutaneous preadipocytes were downloaded from NCBI (accession number: SRX4646736).

### Data Analysis of ISO-Seq Raw Data

We obtained all raw data and processed it according to the Iso-seq standard pipeline^1^. Firstly, the sequence adapters were removed and the sequences shorter than 300 bp in length and less than 0.75 accuracy were filtered to obtain subreads. After quality control, the clean polymerase reads were processed to separate reads of an insert with pass >0 and accuracy >0.75. These reads of insert (ROI) were categorized into full-length, non-full-length, and chimeric reads using the SMRT Iso-Seq analysis pipeline. Full-length reads were determined by detecting poly(A) tails, 5′ primers and 3′ primers. ROI was divided into chimeric transcripts and non-chimeric transcripts according to whether there were sequencing primers in the sequence. The cd-hit-est (Li and Godzik, 2006) were used to remove redundant sequences, and all reserved non-redundant sequences are used for downstream analysis.

### Analysis of Transcript Structure and Alternative Splicing Identification

We aligned the non-redundant isoforms to the Mallard reference genome using GMAP (Wu and Watanabe, 2005) software (gmap.avx512), and sorted the aligned sam files and converted it into bam files using Samtools (Pertea et al., 2016) (v1.9). MatchAnnot (2015.06) software was used to compare the sorted alignment files with the annotation files of the Mallard genome. According to the exons information derived from annotation files, the different matching results were scored. The scores were marked into 0, 1, 2, 3, 4, and 5 to show the best matching transcripts.

In order to identify the alternative splicing (AS) events of the genes, firstly, The alignment file was filtered for 90% alignment coverage and 90% alignment identity and corresponding GFF files generated using cDNA_Cupcake (Gordon et al., 2015) (v18.1.0). SUPPA2 (Trincado et al., 2018) (v2.2.1) identified the AS events from annotation files (GFF/GTF format). The AS events were detected by SUPPA2 (v2.2.1), including alternative 5′ splice site or alternative 3′ splice site (A5/A3), skipping exon (SE), first exon/last exon alternative splicing (AF/AL), mutually exclusive exons (MX) and intron retention (RI).

### Functional Annotation of the Full-Length Transcriptome

In order to annotate non-redundant full-length transcripts, we used Blast (McGinnis and Madden, 2004) (v2.2.26; e value = 1-e5) to align the sequences with the NT (NCBI nucleotide sequences), Swiss-Prot (A manually annotated and reviewed protein sequence database), and KOG (Karyotic Ortholog Groups) databases, and the results with the highest alignment score were used as the annotation. At the same time, we used the known transcription factors of human in the AnimalTFDB database to annotate full-length non-redundant sequences, which is essential for understanding the diversity of transcription factors in adipose. We performed GO (Gene Ontology) and KEGG (Kyoto Encyclopedia of Genes and Genome) functional annotations on the Metascape website (Zhou et al., 2019).

### Differentially Expressed Isoforms in Abdominal and Subcutaneous Preadipocytes Cells

The clean reads of abdominal and subcutaneous preadipocytes cells were mapped to the Mallard reference genome (Anas_platyrhynchos.ASM874695v1.dna.toplevel.fa, Ensembl Release 104, CAU-Wild1.0) by Hisat2 (v2.1.0). Then samtools (v1.9) were used to sort and convert the comparison files. Stringtie (Pertea et al., 2016) (v2.0.6) was used to calculate the abundance of full-length transcripts in each sample by annotation files. The DEseq2 (v1.28.1) was used to analyze differentially expressed transcripts (DETs). Transcripts with the fold change >1.5 and FDR < 0.05 were considered DETs.

### RNA Isolation and Real-Time Quantitative RT-PCR

The total RNA of abdominal and subcutaneous preadipocytes were extracted with e.z.n.a. total RNA kit II reaction kit (Omega Bio-Tek, United States). The RNA quality and quantity were determined using 1% agarose gel electrophoresis and NanoDrop 1000 (Thermo Scientific, Wilmington, DE, United States), and 2 μg of total RNA from abdominal and subcutaneous preadipocytes were reverse-transcribed with PrimeScript^TM^ RT reagent Kit with gDNA Eraser reaction kit (Takara bio, CA, United States) (Kang et al., 2017). Using total RNA as template and oligo (DT) primer, the first strand of cDNA was inverted. The specific primer pairs of transcripts were designed using the Primer-BLAST software^2^. We tested different annealing temperatures to optimize each pair of primers using conventional PCR to exclude the presence of unspecific products or primer dimer synthesis; the PCR products were analyzed by 2% agarose gel electrophoresis. Real-time fluorescence quantification PCR (RT-qPCR). Real-time fluorescence quantification PCR (RT-qPCR) was performed using TB green premix Ex Taq^TM^ fluorescence quantitative kit (Takara, CA, United States) and 7500 Fast Real-Time PCR system (Applied Biosystems, v2.0.6). Each qPCR reaction had a final volume of 20 μL of the reaction mixture, which consisted of 10 μL 2X TB Green Premix Ex Taq, 0.4 μL ROX Reference DyeII, 6.8 μL DNase/RNase-Free water, 0.4 μL forward and reverse specific primers for each transcript and 2 μL of cDNA template (Madkour et al., 2021). The PCR reaction conditions were pre denaturation at 95°C for 30 s, using 40 cycles (95°C for 5 s and 60°C for 30 s), and each sample was technically repeated three times. Fluorescence data were acquired at the end of the extension step. The primer sequences used in RT-qPCR reaction are shown in Supplementary Table S5. Results of the data were obtained by 7500 fast Real-Time PCR system. GAPDH was used as the internal reference gene in each sample to standardize the expression level of the transcripts, and the relative expression was calculated by 2^–ΔΔCT^ relative quantitative method.

## Results

### Phenotypic Difference of Abdominal and Subcutaneous Preadipocytes in Pekin Duck

**FIGURE 1:**
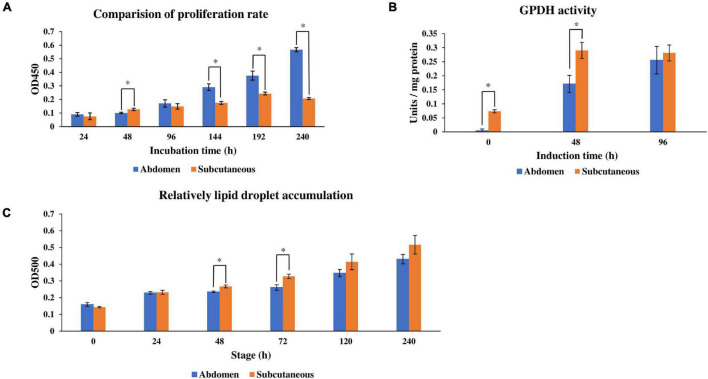
Proliferation rate, GPDH activity, and intracellular lipid droplet accumulation of abdominal adipose and subcutaneous adipose of ducks cultured in differentiation medium (induction). **(A)** Relative quantification of cell proliferation rate within 24–240 h after induction. **(B)** Analysis of GPDH activity at 0 h, 48 h, and 96 h after induction. **(C)** Relative quantification of lipid droplet accumulation within 240 h after induction. The line graph represents the SD of the average (*n* = 3). *Indicates that there is a statistically significant difference between abdominal and subcutaneous preadipocytes at the same time (*P* < 0.05). The statistics data of subcutaneous preadipocytes in panels **(B,C)** were cited by Wang et al. (2019).

Adipogenesis includes the proliferation and differentiation of preadipocytes. In order to compare the phenotypic differences between abdominal and subcutaneous preadipocytes in Pekin duck, the proliferation and differentiation ability of abdominal and subcutaneous preadipocytes in different stages were determined. CCK8 cell proliferation assay results showed that the proliferation rate of abdominal preadipocytes was higher than subcutaneous preadipocytes at 96 h, and the number of living cells in the abdominal preadipocytes group was significantly higher than that of subcutaneous preadipocytes at 144 h, 192 h, and 240 h (*P* < 0.05) (Figure 1A). These results show that the proliferation ability of abdominal preadipocytes was significantly stronger than subcutaneous preadipocytes. GPDH activity assay showed that GPDH activity of subcutaneous preadipocytes was significantly higher than abdominal preadipocytes at 0 h and 48 h (*P* < 0.05) (Figure 1B). Meanwhile, the relative lipid droplet content of subcutaneous and abdominal preadipocytes at different time points after induction showed that the lipid droplet content of subcutaneous preadipocytes was higher than abdominal preadipocytes at each stage, and the difference was significant at 48 h and 72 h (*P* < 0.05) (Figure 1C). These results indicate that the differentiation ability of subcutaneous preadipocytes is stronger than abdominal preadipocytes.

### Generating Transcript Isoforms in Duck Adipose Tissues

**FIGURE 2:**
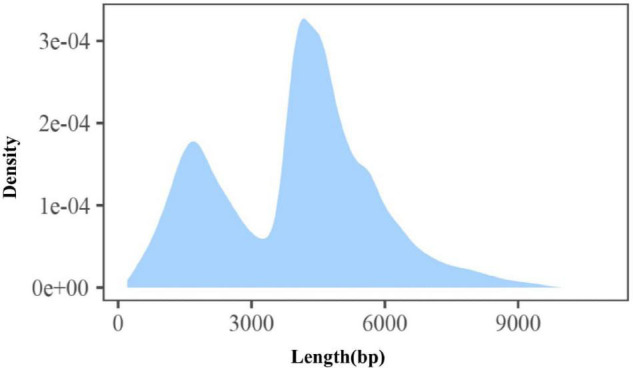
Length distribution of full-length non-redundant transcript.

In order to develop a comprehensive catalog of transcript isoforms, size-fractionated libraries (1–10 kb and 4–10 kb) were constructed. Combining the two libraries, a total of 41.78 GB of raw data was obtained, of which the raw data of abdominal and subcutaneous adipose were both 20.89 GB. For the sequencing data, after filtering and quality control, the number of circulating consensus sequences (CCS) of abdominal and subcutaneous adipose were 240,094 and 184,457, with an average length of 3,858 bp and 3,692 bp. As expected, the average length of these ROI (reads of insert) were consistent with the selected library size. In the study, we classified ROI and obtained full-length non-chimeric transcripts and non-full-length non-chimeric transcripts. 143,931 (59.95%) and 111,337 (60.36%) full-length non-chimeric transcripts of abdominal and subcutaneous adipose were obtained respectively (Table 1). The full-length non-chimeric transcripts of abdominal and subcutaneous adipose were combined and subjected to cluster, and 89,289 full-length non-redundant sequences were obtained, with an average length of 3,985 bp (Figure 2). All full-length non-redundant sequences were used for downstream analysis (Supplementary Figure S9).

**TABLE 1:** Full length transcript data information.

Process	Information	Abdominal	Subcutaneous
Sequencing data output	Cell number	1	1
	cDNA size	1–10 k, 4–10 k	1–10 k, 4–10 k
	Polymerase read bases	20.89	20.89
	Polymerase reads	545895	545895
	Polymerase read N50	68983	68983
	Polymerase read length	38266	38266
ROI	cDNA size	1–10 k	1–10 k
	Reads of insert	240094	184457
	Read bases of insert	926290963	681050538
	Mean read length of insert	3858	3692
	Mean read quality of insert	0.9775004	0.9783864
	Mean number of passes	15	16
Classify	cDNA size	1–10 k	1–10 k
	Number of reads of insert	240094	184457
	Number of five–five prime reads	7347	4919
	Number of three–three prime reads	79460	60854
	Number of adaptor reads	65	81
	Number of filtered short reads	65	81
	Number of full-length non-chimeric reads	143931	111337
			

### Identify the Complex Isoforms of Transcription Factors and Long Non-coding RNAs

**FIGURE 3:**
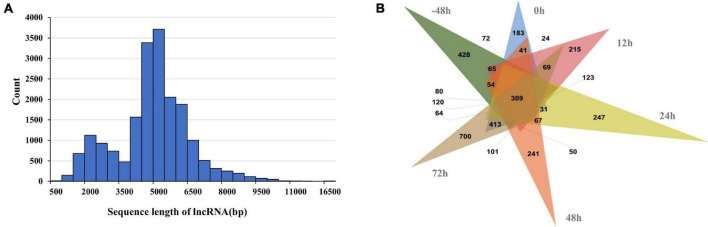
**(A)** Length distribution and full-length transcripts of lncRNAs in abdominal and subcutaneous adipose. **(B)** The number of DET of lncRNA in abdominal adipose and subcutaneous at each time point.

The transcription factor annotation of Mallard duck in AnimalTFDB3.0 database contains 865 annotated transcription factors from 71 families. The 4,544 full-length non-redundant transcripts in our study were annotated 527 transcription factors which belong to 64 gene families (Supplementary Table S1). In most eukaryotes, long-non-coding RNAs (lncRNAs) play an important role in regulating the protein-coding gene expression. In the study, we evaluated the coding potential of the transcripts and obtained 35,134 potential lncRNAs. Then we filtered the transcripts which have high similarity to the known protein sequences. Finally, 19,212 high-confidence lncRNAs were obtained. The length of most of the lncRNAs was 4,000–6,500 bp (Figure 3A), with an average length of 4,469 bp, which is significantly longer than the known length of lncRNAs (1,284 bp). Because lncRNAs is involved in a variety of cellular processes, the diversity and expression difference (Figure 3B) of lncRNAs reflects the complexity of regulatory processes in adipose tissue.

### Structure Annotation

**FIGURE 4:**
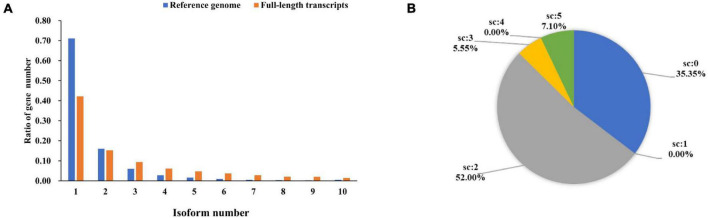
**(A)** Quantity distribution of mallard duck reference genome and full-length non-redundant transcripts. **(B)** Proportional distribution of structural annotations of full-length transcripts. (Score = 0, isoform overlaps gene, but little or no exon congruence; Score = 1, Some exons overlap, overlaps are 1-for-1 where they exist; Score = 2, The best match among all transcripts with score of 1; Score = 3, One-for-one exon match, but sizes of internal exons disagree; Score = 4, Like 5, but leading and trailing edge sizes differ by a larger amount than the score-5 transcript found for this gene; Score = 5, Iso-Seq exons match annotation exons one-for-one. Sizes agree except for leading and trailing edges).

In this study, the amount of multi-exon transcripts were sequenced using PacBio Iso-Seq. At the isoform level, there were 29,320 transcripts from 18,490 wild duck gene models. By comparing with wild duck reference annotation, 62,469 full-length non-redundant transcripts were identified from 11,834 Pekin duck gene models. We found that 71.15% of the genes in the original annotation were defined by a single transcript isoform. After analysis of the Iso-Seq data, only 42.21% of the genes are defined by only a single transcript. The percentage of gene models with at least three isoforms in the full-length non-redundant transcripts was higher than that in the reference transcripts (42.52% vs. 12.83%). On average, there were 5.28 transcripts per gene, compared with 1.59 transcripts in the reference notes (Figure 4A). In addition, the full-length non-redundant sequences were annotated in the study. The different scores in the annotation results correspond to the consistency with the known reference transcripts (Figure 4B). The sequences with matching degree < 2 were poor matching, accounting for 35.35%, while the sequences with potential alternative splicing (score ≥ 2) accounted for 64.66%. These results indicate that the full-length non-redundant transcripts can predict new transcripts based on known transcripts. The exons differences in the number and structure of transcripts show that the AS increases the transcriptome complexity significantly in Pekin ducks.

### Identification of Alternative Splicing Events Based on Mallard Genome

**FIGURE 5:**
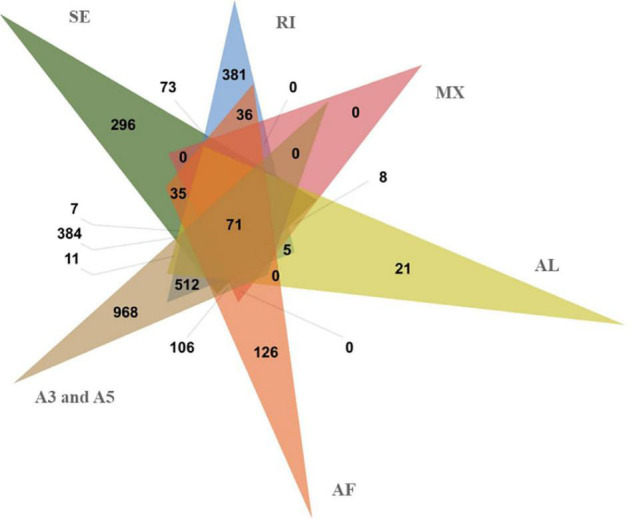
Alternative splice event distribution of abdominal and subcutaneous adipose.

Alternative splicing can increase protein diversity by changing the protein structure. In the full-length non-redundant sequences, 74,571 AS events were identified from 4,046 gene models (Figure 5 and Table 2). The 3′ and 5′ AS (A3/A5) were the main AS events, accounting for 66.59%. The rest AS events included RI (11.80%), SE (5.89%), AF (8.87%), Al (4.51%), and MX (2.33%). A3, A5, IR, and SE AS events are common in genes. Most genes have only one AS type, while only 71 genes have each type. We found that AS events are correlated with the number of exons. With the increase of exons, AS enhanced the diversity and complexity of abdominal and subcutaneous adipose transcripts in Pekin duck.

**TABLE 2:** The number of alternative splicing (AS) event.

Items	Gene counts (%)	Event counts (%)
A3	2064(51.01%)	23873(32.01%)
A5	2283(56.43%)	25788(34.58%)
AF	848(20.96%)	6611(8.87%)
AL	134(3.31%)	3362(4.51%)
MX	198(4.89%)	1742(2.33%)
RI	1906(47.11%)	8802(11.80%)
SE	1733(42.83%)	4393(5.89%)
Total	4046	74571

*A3, alternative 3′ splice site; A5, alternative 5′ splice site; AF, alternative first exon; AL, alternative last exon; MX, mutual exclusive exons; RI, intron retention; SE, exon skipping.*

### Functional Annotation of Transcript Isoforms

**FIGURE 6:**
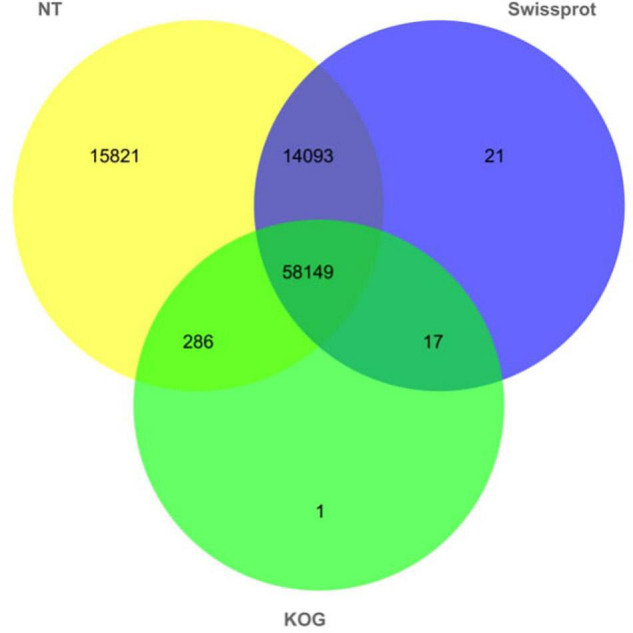
Number distribution of functional notes of full-length non-redundant transcripts of abdominal and subcutaneous adipose in NT, KOG, and Swiss-Prot databases.

In order to obtain the annotation of the full-length transcripts, we annotated full-length transcripts of abdominal and subcutaneous adipose of Pekin duck by NT, Swiss-Prot, and KOG databases for further study of gene function (Figure 6, Supplementary Table S2, and Supplementary Figures S1, S2). In NT, Swiss-Prot, and KOG databases, at least 88,388 (98.99%) transcripts were annotated from 89,289 full-length transcripts. The results showed that 88,349 (98.94%) transcripts were annotated in NT database, and 58,149 (65.43%) transcripts were annotated in all databases. The above results show the reliability and accuracy of the full-length transcripts. All genes corresponding to full-length transcripts were subjected to functional annotation, about 9,495 genes were annotated by GO and KEGG. In GO and KEGG databases, the majority gene symbols of abdominal and subcutaneous adipose were represented by protein binding (671), nucleoplasm (379), and protein ubiquitination (42) in ‘Molecular Function’ category, ‘Cellular Component’ category, and ‘Biological Process’ category, analysis of the KEGG annotations revealed that most genes were enriched in ‘metabolic’ pathway (72).

### Specific Expression of Isoforms in Different Types of Adipocytes

**FIGURE 7:**
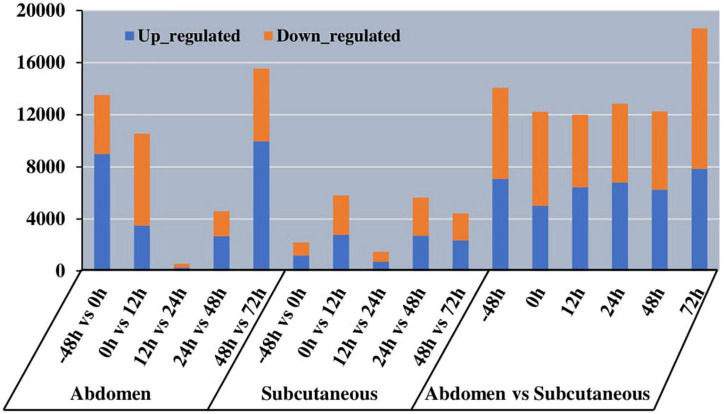
Histogram of the number of different expression transcript (DET) in different tissues at different time of abdominal and subcutaneous adipose.

Our study explored 36 mRNA-Seq libraries to investigate expression level differences between abdominal and subcutaneous adipocytes at six time points. We identified 14054, 12226, 11995, 12854,12255, and 18627 DETs at −48 h, 0 h, 12 h, 24 h, 48 h, and 72 h (Figure 7). The data (Supplementary Table S4) indicate that there may be differences in the number and function of transcriptional regulators before and after differentiation, which reflects the complexity of the regulation mode in the process of Pekin duck fat deposition.

### Discovery of a Specific Transcription Factor During Fat Development

**FIGURE 8:**
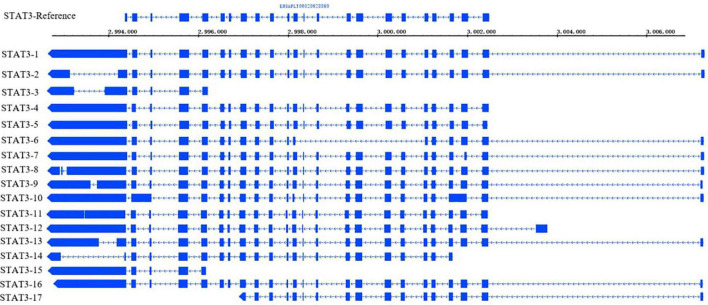
STAT gene transcripts structure diagram.

Many transcription factors play vital roles in the regulatory pathways of preadipocyte STAT3 is signal transduction and transcriptional activator. It is a crucial factor for the biological functions of eukaryotes, such as embryonic development, immunity, hematopoiesis, and cell migration (Liu et al., 2021). STAT3 regulates VSTM2A (V-set and transmembrane domain-containing 2A) (Al Dow et al., 2021) and JAK (Janus kinase) (Zhang et al., 2011) in preadipocytes and promotes white adipose tissue development. According to the results of AS events and annotation of full-length transcripts in the study, STAT3 has 17 isoforms and 4 alternative splicing events, including A5, A3, AF, and RI (Figure 8). The length and number of exons vary among different transcripts. These transcripts contain 5–26 exons, and the transcript with the largest number of exons has two more exons than STAT3 in the reference genome. The number of STAT3 improved the richness of the transcript AS. For example, STAT3-12 contains 24 exons and 4 types of AS. The main AS events of STAT3 are A3 and A5, which involve trans-activated domain and tandem donor reflecting the diversity of transcripts structure.

**FIGURE 9:**
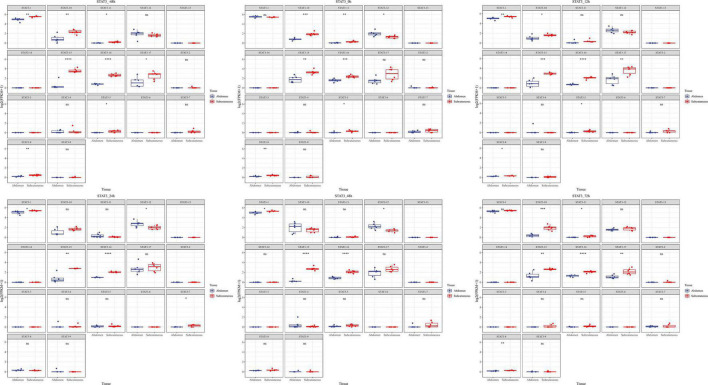
Expression level of new transcripts of STAT gene at different time points of differentiation. Statistical change was determined by Student’s test (two tailed), *P < 0.05; **P < 0.01; ***P < 0.001; ****P < 0.0001 as fat.

**FIGURE 10:**
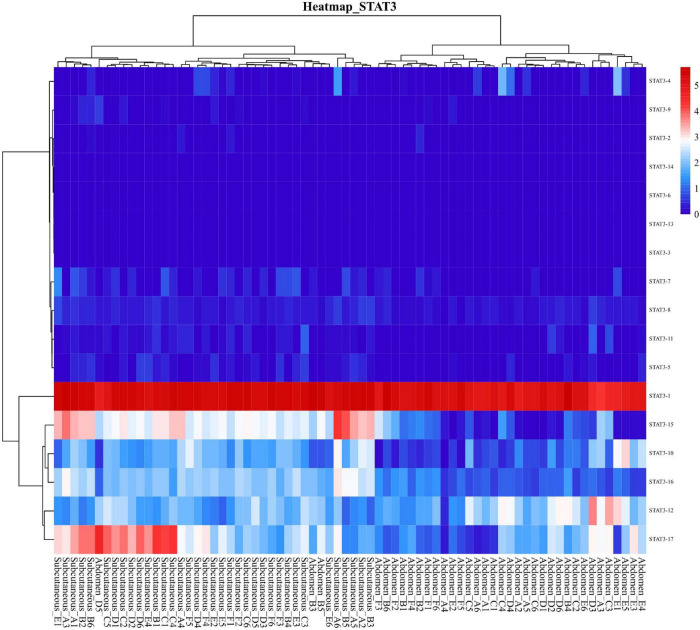
Heatmap of STAT gene transcripts expression.

Total transcripts of STAT3 show various expression patterns during differentiation (Figures 9, 10). STAT3-2, STAT3-3, and STAT3-13 have low-level gene-expression, and STAT3-1 of abdominal and subcutaneous adipocytes are expressed at a considerably higher level than any minor transcripts of the STAT3 during all stages. STAT3-1 has one more exon at the 3′ than the SATA3 in the reference genome. At the early stage of differentiation (12 h, 24 h, and 48 h), the expression level of STAT3-1 in subcutaneous adipocytes was significantly higher than that in abdominal adipocytes (*P* < 0.05). STAT3-10, STAT3-15, STAT3-16, and STAT3-17 have significant difference during preadipocytes differentiation (*P* < 0.05). In abdominal and subcutaneous adipocytes, the expression of STAT3-4, STAT3-8, STAT3-10, STAT3-12, STAT3-16, and STAT3-17 were significantly different at several consecutive stages (*P* < 0.05). These results reflect that the differences in the expression of transcripts lead to the complexity of gene expression among different tissues, which is a potential factor causing the functional complexity of different tissues. Our study analyzed the expression profiles of other transcription factors, such as PPARγ (Supplementary Figures S3, S4), BCL6 (Supplementary Figures S5, S6), and GATA2 (Supplementary Figures S7, S8). The results indicate that the differences in transcript abundance may be mostly attributed to splicing.

### RT-qPCR Assays for Validation of Isoforms

**FIGURE 11:**
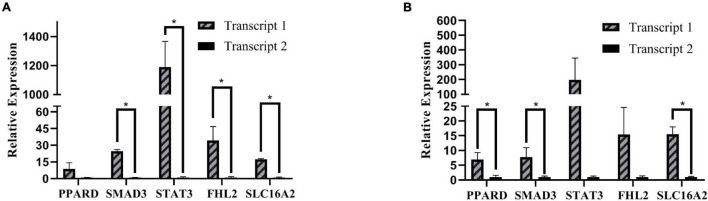
RT-qPCR relative expression. **(A)** Expression of different transcripts of subcutaneous adipose. **(B)** Expression of different transcripts of abdominal adipose, *P < 0.05.

In the current study, three samples of abdominal and subcutaneous preadipocytes from 0 h were randomly selected for RT-qPCR to validate some key genes involved in the proliferation and differentiation of preadipocytes. Including PPARD (Peroxisome Proliferator-Activated Receptor Delta), SMAD3 (SMAD Family Member 3), STAT3, FHL2 (Four and A half LIM domains 2), and SLC16A2 (Solute Carrier Family 16 Member 2) (Figure 11). We choose two transcripts with the highest expression (transcript 1) and lower expression (transcript 2) for each gene. The expression patterns of these transcripts were highly consistent with the mRNA-Seq results. The results showed that transcription factors usually have an obvious dominant expression transcript in adipocytes, which may mainly perform gene functions.

## Discussion

Duck is an important economic waterfowl, and it is also a model animal for adipose deposition and immune research (Li and Lu, 2011; Zheng et al., 2014). Although the duck genome sequence has been released, its information of genome and transcriptome still needs to be further explored. At present, the research related to transcriptome has been reported in ducks. Wang et al. (2019) analyzed the dynamic transcriptome information of the proliferation and differentiation of subcutaneous preadipocytes, Qiu et al. (2018) and Xu et al. (2019) discussed the relationship between gene expression and adipose distribution. These findings lay the foundation for understanding duck fat synthesis and deposition. These studies only paid attention to the expression changes of transcripts, but not reported the above alternative splicing and SNP. Yin et al. (2019) published the full-length transcripts information of the pectoral muscle, heart, uterus, ovary, testis, hypothalamus, pituitary gland, and 13-day-old embryos of Pekin duck, and identified lncRNAs and AS events, which improved genome annotations and informative basis with the functional genomics of other birds. The drawback with this study is that it lacks information about the full-length transcripts of adipose, which hinders the accurate analysis of the gene function related to the adipogenesis of Pekin duck. In our study, Iso-seq analysis was performed on the abdominal and subcutaneous adipocytes of Pekin duck, 62,469 full-length transcripts were identified from 11,834 gene models, transcription factors, lncRNAs, and alternative splicing events were identified to facilitate functional genomics in adipose of Pekin duck. The number of transcription factors identified was less than the total transcription factors of wild ducks, which might be due to some transcripts could not be detected because of the specificity of adipose tissue expression (Rodriguez et al., 2020).

Exploring functional differences requires accurate transcript annotations. Alternative splicing increases the richness of transcripts, which has great significance for functional genomics research. In this study, 74,571 AS events were identified from 4,046 gene models. A5 and A3 involving 2,283 and 2,064 genes had the highest ratio of alternative splicing events. Xu et al. (2016) used the next-generation sequencing to analyze the AS of breast muscle and subcutaneous adipose. Their results showed that A5 and A3 were the main AS events, which were consistent with the results of our study. But the results of this study are different with Yin et al. (2019). Their main AS types are RI and SE. The results of this study and Yin et al. (2019) reflect the differences in the types of AS between adipose tissue and other tissues. AS increases the complexity and diversity of transcripts, which may be the basis for the inheritance and regulation of tissues and organs performing different functions. The AS events identified in this study provide clues for subsequent studies, such as tissue-specific expression, a difference of homologous genes function in different tissues, and so on.

Transcription factors play an important regulatory role in the process of adipogenesis. As an important transcription factor, STAT3 can be activated under the action of IL-11 (interleukin-11) to promote the proliferation and migration of mouse adipose mesenchymal stem cells (Yang et al., 2020), and can synergistic effect with HMGA2 (High Mobility Group AT-Hook 2) to promote the process of adipogenesis in mice (Yuan et al., 2017). In humans, STAT3 can activate the expression of CD36 (CD36 molecule) and promote preadipocyte differentiation and lipid deposition (Rozovski et al., 2018; Su et al., 2020). In poultry, the JAK-STAT signaling pathway regulates the proliferation and apoptosis of chicken chondrocytes (Chen et al., 2021) and embryo development (Zhang et al., 2017), and STAT3 affects the angiogenesis of chorioallantoic membrane in female chicken embryos by mediating the VEGF/NO (vascular endothelial growth factor/nitric oxide) pathway (Su et al., 2012). STAT3 encodes different transcripts (Qi and Yang, 2014), and it was found that STAT3α2 generated by exon skipping may play a major role in STAT3 signal transduction on grass carp, which is consistent with the function of STAT3α1 (Du L. et al., 2017). In our study, STAT3-1 was the major expression transcript, which was generated by A3 and A5 alternative splicing. Studies have shown that STAT3 can affect preadipocyte differentiation by regulating the activity of the PPARγ promoter and regulate the process of adipogenesis (Su et al., 2020). The specific function of STAT3-1 needs to be further confirmed. For example, the three exons at the 3′ ends of STAT3 of the reference genome belong to the nitrogen-terminal domain affecting protein interaction (Schwalie et al., 2018). Compared with the reference genome, there is an extra exon at the 3′ ends of STAT3 in full-length transcripts, which may affect the binding affinity to the target genes. The expression of STAT3-1 of Pekin duck’ s subcutaneous at 12 h, 24 h, and 48 h was higher than that of abdominal fat (*P* < 0.05), which may be one of the factors affecting the differentiation ability of subcutaneous than abdominal preadipocytes. Therefore, in this study, A3 of STAT3-1 was a potential key AS event in the regulation of preadipocyte differentiation. The transcription factors can regulate the binding of other genes, promote or inhibit the differentiation of preadipocytes, but the binding efficiency of different transcripts of the same gene is also different. Alternative splice-form plays a key role in the process of gene regulation by generating different transcripts. Further studies can be carried out through knockdown, knockout, and overexpression experiments to explore the specific functions of different transcripts in adipogenesis.

Previous studies showed that abdominal and subcutaneous adipose have different transcriptional characteristics (Burl et al., 2018; Schwalie et al., 2018; Merrick et al., 2019). The process of adipogenesis is characterized by changes in cell morphology (Sebo and Rodeheffer, 2019). The shape of the cells evolves from flat to round cells with triacylglycerol-rich lipid droplets. The material and energy requirements for cytoskeleton reorganization and accumulation of lipids are necessary (Del et al., 2019; Calvo and Izquierdo, 2021; Zhang et al., 2021). Cytoskeleton is composed of protein fibers, which is consistent with the protein binding process in our function annotation study. Subcutaneous and abdominal adipose mediate the adipose synthesis and immune regulation, such as fibrosis, lipid deposition, angiogenesis, and inflammation (Caputo et al., 2021). The above processes are closely related to metabolism, which is consistent with the KEGG annotation results of our study.

In summary, Iso-seq and RNA-Seq conducted a global analysis for the differentiation process of the abdominal and subcutaneous preadipocytes in Pekin duck. This study provided full-length transcripts, AS events, lncRNAs and transcription factors, analyzed expression levels at different stages of preadipocyte differentiation, and initially explored the regulation of AS of abdominal and subcutaneous adipose in Pekin duck. These results definitively provide valuable information for the alternative splicing, gene expression and regulation of adipose tissue in Pekin duck. Furthermore, the information generated will promote future investigations of functional genomics.

## Data Availability Statement

The datasets presented in this study can be found in online repositories. The names of the repository/repositories and accession number(s) can be found below: NCBI BioProject PRJNA723918/SRX4646736.

## Ethics Statement

The animal study was reviewed and approved by Committee of China Agricultural University.

## Author Contributions

ZH conceived and designed the experimental plan. DS and XL collected samples and measurement data. DS and ZY participated in bioinformatics analyses. DS, XL, ZY, and ZH drafted and revised this manuscript. All authors read and approved the final manuscript.

## Conflict of Interest

The authors declare that the research was conducted in the absence of any commercial or financial relationships that could be construed as a potential conflict of interest.

## Publisher’s Note

All claims expressed in this article are solely those of the authors and do not necessarily represent those of their affiliated organizations, or those of the publisher, the editors and the reviewers. Any product that may be evaluated in this article, or claim that may be made by its manufacturer, is not guaranteed or endorsed by the publisher.

## Footnotes

1
https://github.com/PacificBiosciences/IsoSeq_SA3nUP/wiki/Tutorial:-Installing-and-Running-Iso-Seq-3-using-Conda

2
www.ncbi.nlm.nih.gov/tools/primer-blast

## Abbreviations

Iso-Seq: isoform sequencing
AS: alternative splicing
PacBio: Pacific Biosciences
TFs: transcription factors
lncRNAs: long non-coding RNAs
GPDH: glycerol-3-phosphate dehydrogenase
KEGG: Kyoto Encyclopedia of Genes and Genomes
A3: alternative 3′ splice site
A5: alternative 5′ splice site
AF: alternative first exon
AL: alternative last exon
MX: mutual exclusive exon
RI: intron retention
SE: exon skipping
PPAR γ: peroxisome proliferative activated receptor, gamma
STAT3: signal transducer and activator of transcription 3.
