# Supplementary material for: The Full-Length Transcriptome Provides New Insights Into the Transcript Complexity of Abdominal Adipose and Subcutaneous Adipose in Pekin Ducks
Source: Front Physiol. 2021 Nov 10;12:767739. doi: 10.3389/fphys.2021.767739 (PMC8631521; doi:10.3389/fphys.2021.767739)
Supplement: Supplementary file 1 [file Data_Sheet_1.docx]

Supplementary Material


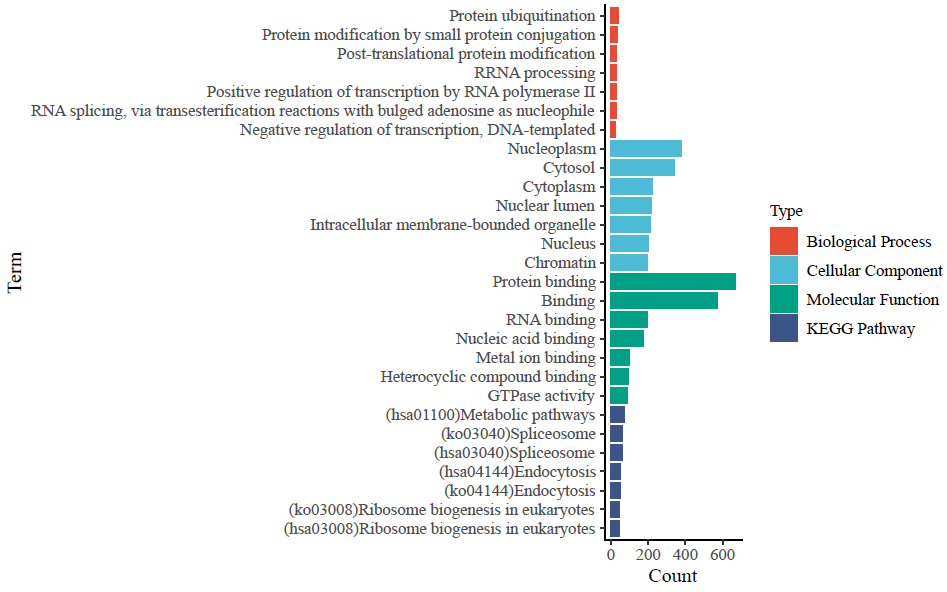


**Figure S1. Go annotation for abdominal and subcutaneous fat.**


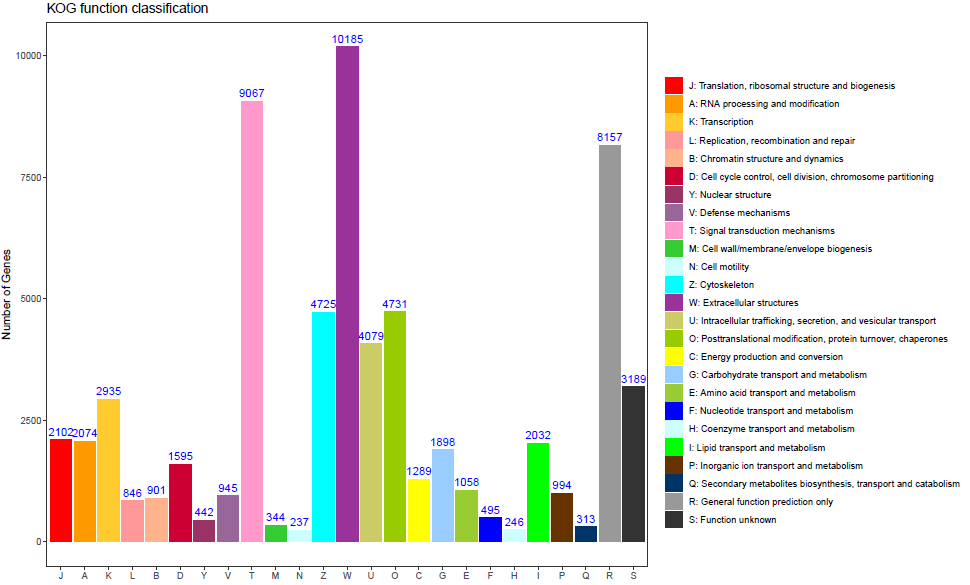


**Figure S2. KOG function annotation bar graph**

**Figure S3. PPARG transcript structure diagram.**


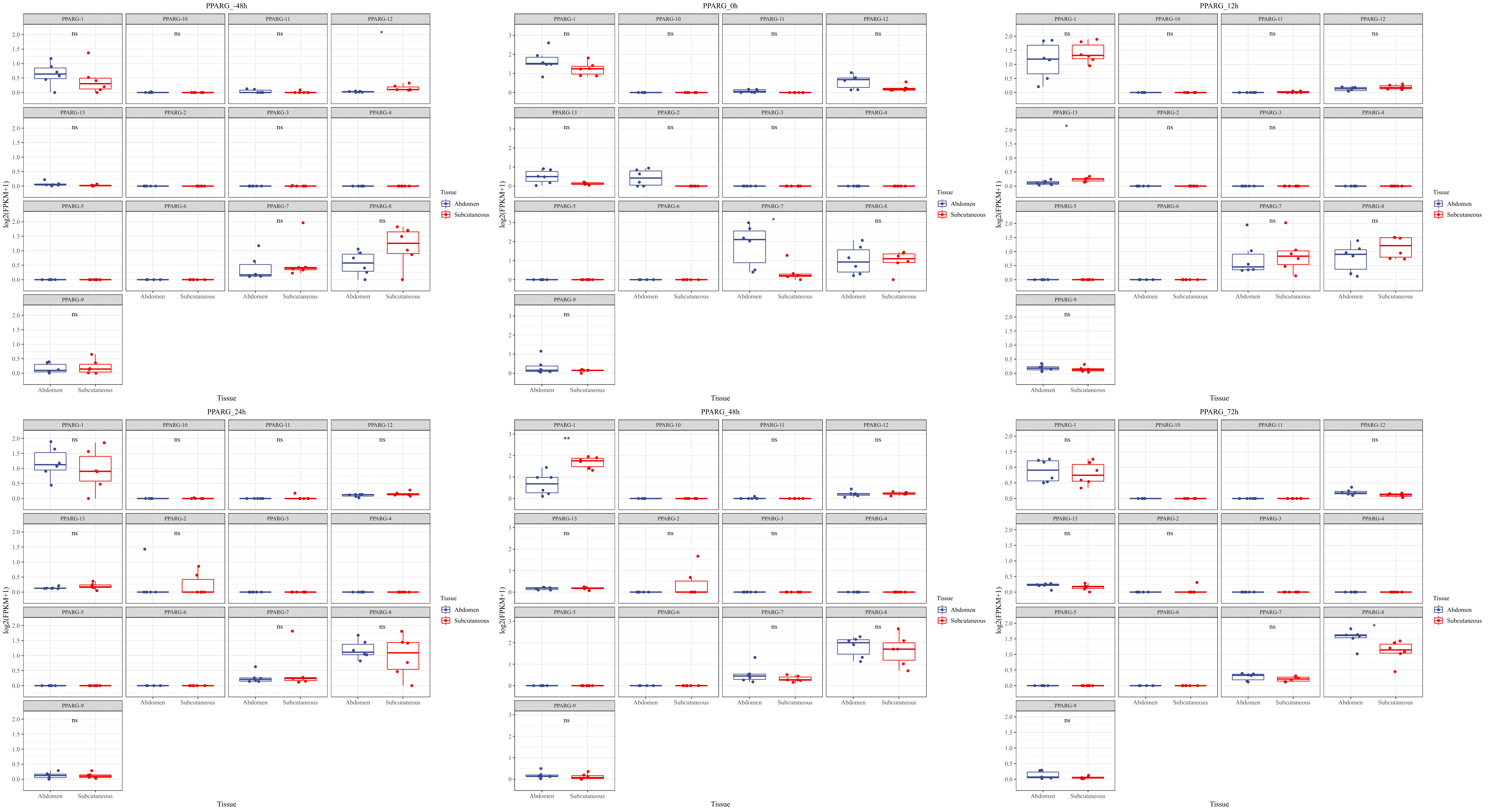


**Figure S4. The expression levels of PPARG transcripts at different time points of differentiation.** The statistical change is determined by Student’s test, *P* < 0.05 is represented as *, *P* < 0.01 is represented as **, *P* < 0.0001 is represented as ****.


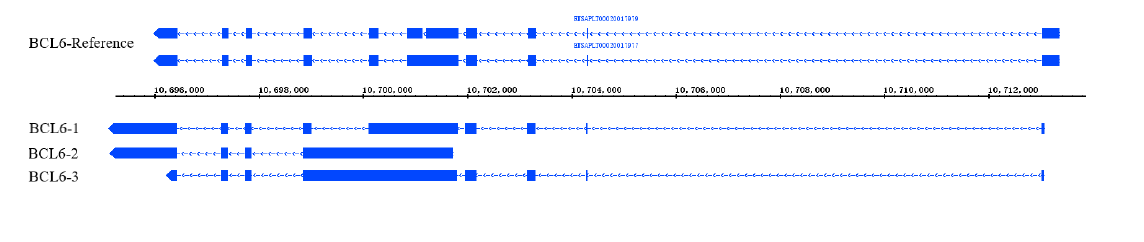


**Figure S5. BCL6 transcript structure diagram.**


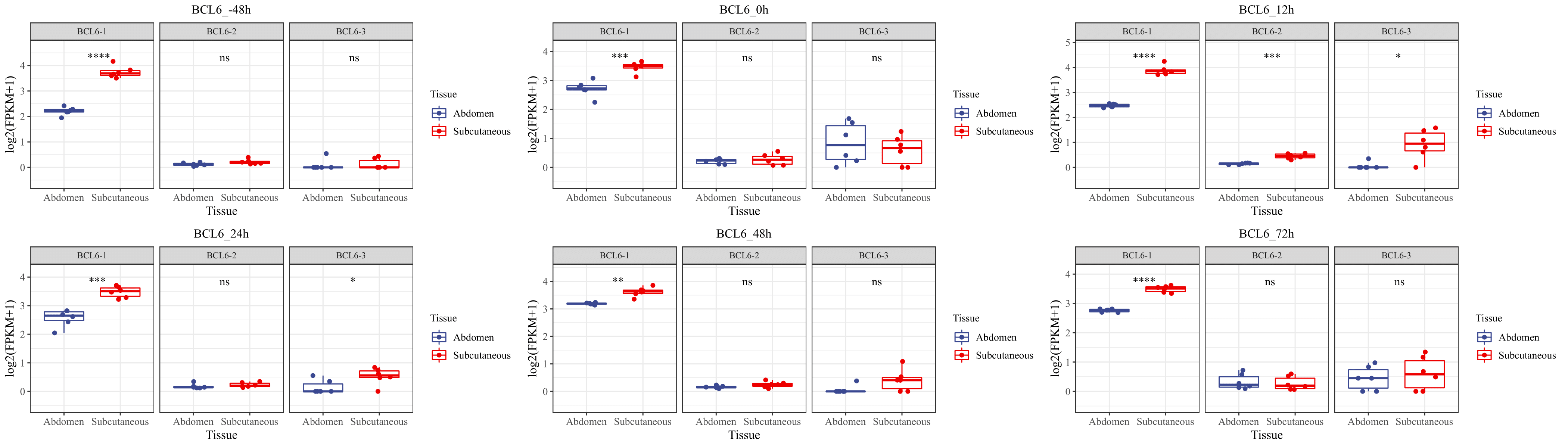


**Figure S6. Expression levels of BCL6 transcripts at different time points of differentiation.** The statistical change is determined by Student’s test, *P* < 0.05 is represented as*, *P* < 0.01 is represented as **, *P* < 0.0001 is represented as ****.


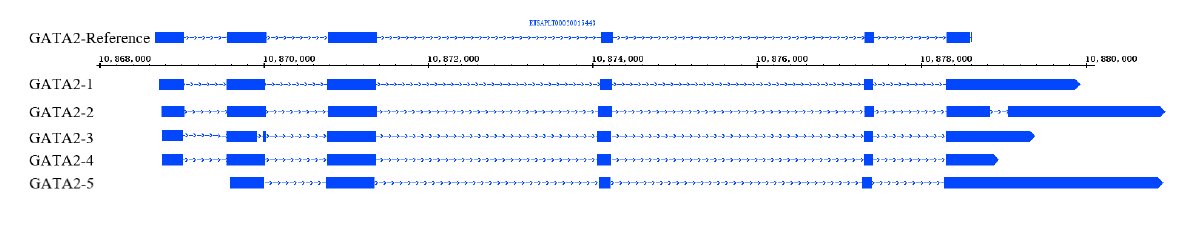


**Figure S7. GATA2 transcript structure diagram**


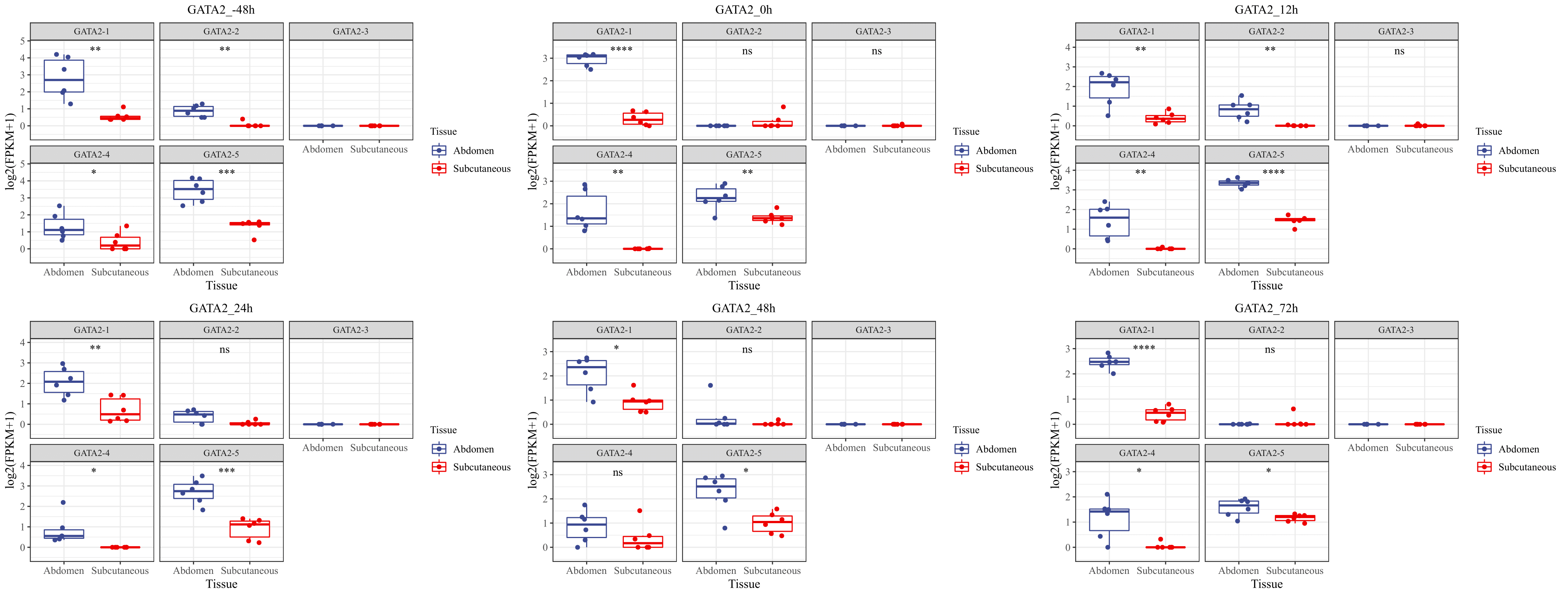


**Figure S8. The expression levels of GATA2 transcripts at different time points of differentiation.** The statistical change is determined by Student’s test, *P* < 0.05 is represented as *, *P* < 0.01 is represented as **, *P* < 0.0001 is represented as ****.

**Figure S9. Full length analysis Roadmap**

**Table S1. Transcription factors (TFs) identified list**

| **Number** | **Gene name** | **Gene family** |
| --- | --- | --- |
| 1 | AFF4 | AF-4 |
| 2 | AFF1 | AF-4 |
| 3 | TFAP2C | AP-2 |
| 4 | ARID2 | ARID |
| 5 | ARID1A | ARID |
| 6 | ARID4B | ARID |
| 7 | ARID4A | ARID |
| 8 | ARID3B | ARID |
| 9 | ARID5B | ARID |
| 10 | ARNT | bHLH |
| 11 | CLOCK | bHLH |
| 12 | HIF1A | bHLH |
| 13 | ARNT2 | bHLH |
| 14 | ARNTL2 | bHLH |
| 15 | NCOA1 | bHLH |
| 16 | TCF4 | bHLH |
| 17 | SREBF2 | bHLH |
| 18 | MITF | bHLH |
| 19 | AHRR | bHLH |
| 20 | MLX | bHLH |
| 21 | MLXIP | bHLH |
| 22 | TFEC | bHLH |
| 23 | ARNTL | bHLH |
| 24 | MXI1 | bHLH |
| 25 | MXD4 | bHLH |
| 26 | MYC | bHLH |
| 27 | ID2 | bHLH |
| 28 | ID3 | bHLH |
| 29 | MNT | bHLH |
| 30 | HAND1 | bHLH |
| 31 | NPAS2 | bHLH |
| 32 | SIM2 | bHLH |
| 33 | CBFB | CBF |
| 34 | EBF3 | COE |
| 35 | EBF2 | COE |
| 36 | EBF1 | COE |
| 37 | TFCP2 | CP2 |
| 38 | TFCP2L1 | CP2 |
| 39 | UBP1 | CP2 |
| 40 | CSDE1 | CSD |
| 41 | YBX1 | CSD |
| 42 | CARHSP1 | CSD |
| 43 | YBX3 | CSD |
| 44 | RBPJ | CSL |
| 45 | CSRNP1 | CSRNP_N |
| 46 | NFIB | CTF/NFI |
| 47 | NFIA | CTF/NFI |
| 48 | CUX1 | CUT |
| 49 | SATB2 | CUT |
| 50 | CUX2 | CUT |
| 51 | DACH1 | DACH |
| 52 | E2F7 | E2F |
| 53 | E2F6 | E2F |
| 54 | E2F3 | E2F |
| 55 | TFDP2 | E2F |
| 56 | E2F4 | E2F |
| 57 | NR3C2 | ESR-like |
| 58 | ESRRG | ESR-like |
| 59 | NR3C1 | ESR-like |
| 60 | ESRRB | ESR-like |
| 61 | AR | ESR-like |
| 62 | ETV1 | ETS |
| 63 | GABPA | ETS |
| 64 | FLI1 | ETS |
| 65 | ERG | ETS |
| 66 | ETV6 | ETS |
| 67 | ETV4 | ETS |
| 68 | ELK3 | ETS |
| 69 | SPI1 | ETS |
| 70 | ELF1 | ETS |
| 71 | ETV5 | ETS |
| 72 | ETS1 | ETS |
| 73 | ELK4 | ETS |
| 74 | ELF2 | ETS |
| 75 | FOXM1 | Fork_head |
| 76 | FOXP4 | Fork_head |
| 77 | FOXN2 | Fork_head |
| 78 | FOXO4 | Fork_head |
| 79 | FOXO3 | Fork_head |
| 80 | FOXO1 | Fork_head |
| 81 | FOXJ2 | Fork_head |
| 82 | FOXK2 | Fork_head |
| 83 | FOXN3 | Fork_head |
| 84 | FOXP2 | Fork_head |
| 85 | TFIP11 | GCFC |
| 86 | PAXBP1 | GCFC |
| 87 | GTF2IRD1 | GTF2I |
| 88 | GTF2I | GTF2I |
| 89 | KMT2C | HMG |
| 90 | PBRM1 | HMG |
| 91 | WDHD1 | HMG |
| 92 | HMGXB3 | HMG |
| 93 | NSD2 | HMG |
| 94 | SSRP1 | HMG |
| 95 | HBP1 | HMG |
| 96 | BBX | HMG |
| 97 | TOX | HMG |
| 98 | SOX5 | HMG |
| 99 | SMARCE1 | HMG |
| 100 | SOX6 | HMG |
| 101 | HMG20A | HMG |
| 102 | HMG20B | HMG |
| 103 | HMGXB4 | HMG |
| 104 | TCF7L2 | HMG |
| 105 | TFAM | HMG |
| 106 | HMGB3 | HMG |
| 107 | HMGB2 | HMG |
| 108 | PMS1 | HMG |
| 109 | HMGB1 | HMG |
| 110 | SOX11 | HMG |
| 111 | HMGA2 | HMGA |
| 112 | ZFHX4 | Homeobox |
| 113 | ADNP | Homeobox |
| 114 | ZHX2 | Homeobox |
| 115 | HDX | Homeobox |
| 116 | SIX4 | Homeobox |
| 117 | PKNOX1 | Homeobox |
| 118 | HMBOX1 | Homeobox |
| 119 | MEIS2 | Homeobox |
| 120 | PBX1 | Homeobox |
| 121 | ZHX1 | Homeobox |
| 122 | HOXA3 | Homeobox |
| 123 | MEIS1 | Homeobox |
| 124 | TGIF1 | Homeobox |
| 125 | MSX2 | Homeobox |
| 126 | ZHX3 | Homeobox |
| 127 | PBX4 | Homeobox |
| 128 | EMX2 | Homeobox |
| 129 | PRRX1 | Homeobox |
| 130 | LHX8 | Homeobox |
| 131 | HOXB7 | Homeobox |
| 132 | HOXA5 | Homeobox |
| 133 | HOXD8 | Homeobox |
| 134 | HOXA7 | Homeobox |
| 135 | HOXC10 | Homeobox |
| 136 | MEOX2 | Homeobox |
| 137 | HOPX | Homeobox |
| 138 | HOXB4 | Homeobox |
| 139 | PKNOX2 | Homeobox |
| 140 | LHX9 | Homeobox |
| 141 | LHX4 | Homeobox |
| 142 | PBX3 | Homeobox |
| 143 | LHX1 | Homeobox |
| 144 | LMX1B | Homeobox |
| 145 | HSF2 | HSF |
| 146 | LCORL | HTH |
| 147 | LCOR | HTH |
| 148 | IRF8 | IRF |
| 149 | IRF7 | IRF |
| 150 | IRF2 | IRF |
| 151 | IRF6 | IRF |
| 152 | LRRFIP2 | LRRFIP |
| 153 | LRRFIP1 | LRRFIP |
| 154 | SETDB1 | MBD |
| 155 | BAZ2B | MBD |
| 156 | SETDB2 | MBD |
| 157 | MBD2 | MBD |
| 158 | SMAD4 | MH1 |
| 159 | SMAD3 | MH1 |
| 160 | SMAD1 | MH1 |
| 161 | SMAD5 | MH1 |
| 162 | SMAD9 | MH1 |
| 163 | NCOR1 | MYB |
| 164 | SMARCA1 | MYB |
| 165 | SNAPC4 | MYB |
| 166 | SMARCA5 | MYB |
| 167 | MYSM1 | MYB |
| 168 | MYBL2 | MYB |
| 169 | CDC5L | MYB |
| 170 | MIER2 | MYB |
| 171 | DMTF1 | MYB |
| 172 | TADA2A | MYB |
| 173 | SMARCC1 | MYB |
| 174 | MIER1 | MYB |
| 175 | RCOR3 | MYB |
| 176 | RCOR1 | MYB |
| 177 | TERF2 | MYB |
| 178 | DNAJC2 | MYB |
| 179 | DNAJC1 | MYB |
| 180 | MYB | MYB |
| 181 | MYBL1 | MYB |
| 182 | MYRF | NDT80/PhoG |
| 183 | NFYA | NF-YA |
| 184 | NFYB | NF-YB |
| 185 | NFYC | NF-YC |
| 186 | NR4A3 | NGFIB-like |
| 187 | NRF1 | Nrf1 |
| 188 | TP73 | P53 |
| 189 | TP63 | P53 |
| 190 | POU2F1 | Pou |
| 191 | RFX7 | RFX |
| 192 | RFX2 | RFX |
| 193 | NFAT5 | RHD |
| 194 | NFKB1 | RHD |
| 195 | NFATC3 | RHD |
| 196 | NFATC2 | RHD |
| 197 | REL | RHD |
| 198 | NFATC1 | RHD |
| 199 | NFKB2 | RHD |
| 200 | RUNX2 | Runt |
| 201 | RUNX1 | Runt |
| 202 | RUNX3 | Runt |
| 203 | NR2C2 | RXR-like |
| 204 | NR2C1 | RXR-like |
| 205 | RXRG | RXR-like |
| 206 | RXRA | RXR-like |
| 207 | NR2F2 | RXR-like |
| 208 | NR2F6 | RXR-like |
| 209 | GMEB1 | SAND |
| 210 | DEAF1 | SAND |
| 211 | NR5A1 | SF-like |
| 212 | MEF2A | SRF |
| 213 | MEF2C | SRF |
| 214 | STAT3 | STAT |
| 215 | STAT1 | STAT |
| 216 | STAT6 | STAT |
| 217 | MGA | T-box |
| 218 | TBX20 | T-box |
| 219 | TBX18 | T-box |
| 220 | TBX15 | T-box |
| 221 | TEAD4 | TEA |
| 222 | TEAD1 | TEA |
| 223 | BACH1 | TF_bZIP |
| 224 | NFE2L2 | TF_bZIP |
| 225 | NFE2L1 | TF_bZIP |
| 226 | NFIL3 | TF_bZIP |
| 227 | ATF2 | TF_bZIP |
| 228 | CREB3L1 | TF_bZIP |
| 229 | JUN | TF_bZIP |
| 230 | CREB1 | TF_bZIP |
| 231 | CREB3L2 | TF_bZIP |
| 232 | MAFB | TF_bZIP |
| 233 | TEF | TF_bZIP |
| 234 | FOSL2 | TF_bZIP |
| 235 | ATF1 | TF_bZIP |
| 236 | BACH2 | TF_bZIP |
| 237 | ATF3 | TF_bZIP |
| 238 | ATF4 | TF_bZIP |
| 239 | ATF6 | TF_bZIP |
| 240 | MAFK | TF_bZIP |
| 241 | CREM | TF_bZIP |
| 242 | FOS | TF_bZIP |
| 243 | XBP1 | TF_bZIP |
| 244 | CEBPG | TF_bZIP |
| 245 | MAFG | TF_bZIP |
| 246 | AHCTF1 | TF_others |
| 247 | ZNF608 | TF_others |
| 248 | ADNP2 | TF_others |
| 249 | EPAS1 | TF_others |
| 250 | TET2 | TF_others |
| 251 | TMF1 | TF_others |
| 252 | ELMSAN1 | TF_others |
| 253 | PHTF1 | TF_others |
| 254 | NFRKB | TF_others |
| 255 | SMARCAL1 | TF_others |
| 256 | BCLAF1 | TF_others |
| 257 | KIAA1549 | TF_others |
| 258 | CEBPZ | TF_others |
| 259 | ZGPAT | TF_others |
| 260 | GPBP1L1 | TF_others |
| 261 | GABPB2 | TF_others |
| 262 | BLZF1 | TF_others |
| 263 | FUBP1 | TF_others |
| 264 | TFB1M | TF_others |
| 265 | ZNF326 | TF_others |
| 266 | CC2D1B | TF_others |
| 267 | NKRF | TF_others |
| 268 | PHB | TF_others |
| 269 | RFXANK | TF_others |
| 270 | ZNF395 | TF_others |
| 271 | THYN1 | TF_others |
| 272 | YEATS4 | TF_others |
| 273 | CARF | TF_others |
| 274 | GABPB1 | TF_others |
| 275 | ZNF207 | TF_others |
| 276 | CNBP | TF_others |
| 277 | MYEF2 | TF_others |
| 278 | CREBRF | TF_others |
| 279 | LYAR | TF_others |
| 280 | MLLT10 | TF_others |
| 281 | CREBL2 | TF_others |
| 282 | NME3 | TF_others |
| 283 | PLEK | TF_others |
| 284 | MAF1 | TF_others |
| 285 | CAPN15 | TF_others |
| 286 | ZNF706 | TF_others |
| 287 | CASZ1 | TF_others |
| 288 | RAG1 | TF_others |
| 289 | THAP12 | THAP |
| 290 | THAP4 | THAP |
| 291 | PPARG | THR-like |
| 292 | PPARA | THR-like |
| 293 | VDR | THR-like |
| 294 | PPARD | THR-like |
| 295 | RARB | THR-like |
| 296 | THRB | THR-like |
| 297 | NR1H3 | THR-like |
| 298 | NR1D2 | THR-like |
| 299 | TSC22D2 | TSC22 |
| 300 | TSC22D3 | TSC22 |
| 301 | TULP3 | Tub |
| 302 | TUB | Tub |
| 303 | TULP4 | Tub |
| 304 | ZBTB11 | ZBTB |
| 305 | ZBTB20 | ZBTB |
| 306 | ZBTB39 | ZBTB |
| 307 | ZBTB41 | ZBTB |
| 308 | ZBTB24 | ZBTB |
| 309 | HIC2 | ZBTB |
| 310 | ZBTB1 | ZBTB |
| 311 | ZBTB21 | ZBTB |
| 312 | ZBTB37 | ZBTB |
| 313 | ZBTB17 | ZBTB |
| 314 | ZBTB44 | ZBTB |
| 315 | ZBTB33 | ZBTB |
| 316 | BCL6 | ZBTB |
| 317 | ZBTB26 | ZBTB |
| 318 | ZBTB49 | ZBTB |
| 319 | ZBTB18 | ZBTB |
| 320 | ZBTB25 | ZBTB |
| 321 | ZBTB16 | ZBTB |
| 322 | MYNN | ZBTB |
| 323 | ZBTB38 | ZBTB |
| 324 | ZBTB8A | ZBTB |
| 325 | ZBTB46 | ZBTB |
| 326 | ZBTB43 | ZBTB |
| 327 | PATZ1 | ZBTB |
| 328 | ZBTB14 | ZBTB |
| 329 | ZBTB7A | ZBTB |
| 330 | ZBTB34 | ZBTB |
| 331 | ZBTB2 | ZBTB |
| 332 | ZBED4 | zf-BED |
| 333 | ZBED1 | zf-BED |
| 334 | RREB1 | zf-C2H2 |
| 335 | ZNF532 | zf-C2H2 |
| 336 | ZFAT | zf-C2H2 |
| 337 | ZNF521 | zf-C2H2 |
| 338 | ZNF592 | zf-C2H2 |
| 339 | PRDM15 | zf-C2H2 |
| 340 | ZNF217 | zf-C2H2 |
| 341 | HIVEP1 | zf-C2H2 |
| 342 | ZEB2 | zf-C2H2 |
| 343 | TSHZ1 | zf-C2H2 |
| 344 | TSHZ2 | zf-C2H2 |
| 345 | HIVEP3 | zf-C2H2 |
| 346 | GLI3 | zf-C2H2 |
| 347 | RLF | zf-C2H2 |
| 348 | PRDM2 | zf-C2H2 |
| 349 | SALL4 | zf-C2H2 |
| 350 | ZNF687 | zf-C2H2 |
| 351 | PRDM10 | zf-C2H2 |
| 352 | ZNF236 | zf-C2H2 |
| 353 | BNC1 | zf-C2H2 |
| 354 | HIVEP2 | zf-C2H2 |
| 355 | TRERF1 | zf-C2H2 |
| 356 | GLIS3 | zf-C2H2 |
| 357 | ZFPM2 | zf-C2H2 |
| 358 | PRDM1 | zf-C2H2 |
| 359 | MECOM | zf-C2H2 |
| 360 | GLI2 | zf-C2H2 |
| 361 | ZNF143 | zf-C2H2 |
| 362 | ZNF800 | zf-C2H2 |
| 363 | PRDM5 | zf-C2H2 |
| 364 | MTF1 | zf-C2H2 |
| 365 | ZNF438 | zf-C2H2 |
| 366 | ZNF281 | zf-C2H2 |
| 367 | ZNF516 | zf-C2H2 |
| 368 | ZNF618 | zf-C2H2 |
| 369 | ZNF507 | zf-C2H2 |
| 370 | ZNF407 | zf-C2H2 |
| 371 | PLAGL1 | zf-C2H2 |
| 372 | ZNF512 | zf-C2H2 |
| 373 | ZNF518B | zf-C2H2 |
| 374 | ZFP64 | zf-C2H2 |
| 375 | ZNF423 | zf-C2H2 |
| 376 | ZNF148 | zf-C2H2 |
| 377 | ZEB1 | zf-C2H2 |
| 378 | IKZF5 | zf-C2H2 |
| 379 | PRDM4 | zf-C2H2 |
| 380 | ZNF277 | zf-C2H2 |
| 381 | ZNF652 | zf-C2H2 |
| 382 | ZNF827 | zf-C2H2 |
| 383 | ZNF644 | zf-C2H2 |
| 384 | ZNF319 | zf-C2H2 |
| 385 | ZNF292 | zf-C2H2 |
| 386 | SP4 | zf-C2H2 |
| 387 | PLAG1 | zf-C2H2 |
| 388 | ZNF341 | zf-C2H2 |
| 389 | PLAGL2 | zf-C2H2 |
| 390 | HINFP | zf-C2H2 |
| 391 | ZNF410 | zf-C2H2 |
| 392 | ZNF469 | zf-C2H2 |
| 393 | KLF15 | zf-C2H2 |
| 394 | PRDM16 | zf-C2H2 |
| 395 | ZNF622 | zf-C2H2 |
| 396 | ZNF76 | zf-C2H2 |
| 397 | ZIC3 | zf-C2H2 |
| 398 | ZNF384 | zf-C2H2 |
| 399 | OSR1 | zf-C2H2 |
| 400 | ZNF711 | zf-C2H2 |
| 401 | REST | zf-C2H2 |
| 402 | KLF7 | zf-C2H2 |
| 403 | SNAI2 | zf-C2H2 |
| 404 | CTCF | zf-C2H2 |
| 405 | KLF6 | zf-C2H2 |
| 406 | SP3 | zf-C2H2 |
| 407 | EGR1 | zf-C2H2 |
| 408 | ZNF639 | zf-C2H2 |
| 409 | YY1 | zf-C2H2 |
| 410 | KLF3 | zf-C2H2 |
| 411 | OSR2 | zf-C2H2 |
| 412 | WT1 | zf-C2H2 |
| 413 | GZF1 | zf-C2H2 |
| 414 | ZNF692 | zf-C2H2 |
| 415 | SP1 | zf-C2H2 |
| 416 | ZMAT3 | zf-C2H2 |
| 417 | VEZF1 | zf-C2H2 |
| 418 | BCL11B | zf-C2H2 |
| 419 | KLF5 | zf-C2H2 |
| 420 | ZNF512B | zf-C2H2 |
| 421 | ZNF276 | zf-C2H2 |
| 422 | ZNF821 | zf-C2H2 |
| 423 | KLF13 | zf-C2H2 |
| 424 | KLF11 | zf-C2H2 |
| 425 | ZC2HC1B | zf-C2H2 |
| 426 | ZXDC | zf-C2H2 |
| 427 | ZNF142 | zf-C2H2 |
| 428 | ZNF513 | zf-C2H2 |
| 429 | AEBP2 | zf-C2H2 |
| 430 | L3MBTL3 | zf-C2HC |
| 431 | MYT1L | zf-C2HC |
| 432 | TRPS1 | zf-GATA |
| 433 | MTA3 | zf-GATA |
| 434 | MTA1 | zf-GATA |
| 435 | GATAD2A | zf-GATA |
| 436 | RERE | zf-GATA |
| 437 | GATAD2B | zf-GATA |
| 438 | GATA2 | zf-GATA |
| 439 | GATA5 | zf-GATA |
| 440 | GATA6 | zf-GATA |
| 441 | LITAF | zf-LITAF-like |
| 442 | ZMIZ1 | zf-MIZ |
| 443 | PIAS1 | zf-MIZ |
| 444 | PIAS4 | zf-MIZ |
| 445 | NFX1 | zf-NF-X1 |
| 446 | NFXL1 | zf-NF-X1 |
| 447 | ENSAPLP00000003174 | AF-4 |
| 448 | ENSAPLP00000015964 | AF-4 |
| 449 | ENSAPLP00000000926 | AF-4 |
| 450 | ENSAPLP00000007235 | ARID |
| 451 | ENSAPLP00000003831 | ARID |
| 452 | ENSAPLP00000008476 | bHLH |
| 453 | ENSAPLP00000003414 | bHLH |
| 454 | ENSAPLP00000014836 | bHLH |
| 455 | ENSAPLP00000001831 | bHLH |
| 456 | ENSAPLP00000014818 | bHLH |
| 457 | ENSAPLP00000007718 | bHLH |
| 458 | ENSAPLP00000004648 | bHLH |
| 459 | ENSAPLP00000014283 | E2F |
| 460 | ENSAPLP00000008775 | E2F |
| 461 | ENSAPLP00000014950 | ETS |
| 462 | ENSAPLP00000011572 | Fork_head |
| 463 | ENSAPLP00000004310 | GCNF-like |
| 464 | ENSAPLP00000008185 | HMG |
| 465 | ENSAPLP00000013466 | Homeobox |
| 466 | ENSAPLP00000013473 | Homeobox |
| 467 | ENSAPLP00000014026 | Homeobox |
| 468 | ENSAPLP00000005496 | Homeobox |
| 469 | ENSAPLP00000000002 | Homeobox |
| 470 | ENSAPLP00000004696 | HSF |
| 471 | ENSAPLP00000002853 | MBD |
| 472 | ENSAPLP00000007799 | MH1 |
| 473 | ENSAPLP00000007549 | MYB |
| 474 | ENSAPLP00000013463 | MYB |
| 475 | ENSAPLP00000006251 | MYB |
| 476 | ENSAPLP00000011379 | MYB |
| 477 | ENSAPLP00000010591 | MYB |
| 478 | ENSAPLP00000016285 | MYB |
| 479 | ENSAPLP00000009176 | NCU-G1 |
| 480 | ENSAPLP00000015863 | PC4 |
| 481 | ENSAPLP00000000831 | TF_bZIP |
| 482 | ENSAPLP00000015037 | TF_bZIP |
| 483 | ENSAPLP00000003887 | TF_bZIP |
| 484 | ENSAPLP00000012102 | TF_bZIP |
| 485 | ENSAPLP00000010967 | TF_others |
| 486 | ENSAPLP00000014020 | TF_others |
| 487 | ENSAPLP00000006041 | TF_others |
| 488 | ENSAPLP00000006561 | TF_others |
| 489 | ENSAPLP00000003024 | TF_others |
| 490 | ENSAPLP00000014655 | TF_others |
| 491 | ENSAPLP00000001419 | ZBTB |
| 492 | ENSAPLP00000001515 | ZBTB |
| 493 | ENSAPLP00000012008 | ZBTB |
| 494 | ENSAPLP00000004220 | ZBTB |
| 495 | ENSAPLP00000011771 | ZBTB |
| 496 | ENSAPLP00000007938 | zf-C2H2 |
| 497 | ENSAPLP00000013881 | zf-C2H2 |
| 498 | ENSAPLP00000014593 | zf-C2H2 |
| 499 | ENSAPLP00000013390 | zf-C2H2 |
| 500 | ENSAPLP00000009302 | zf-C2H2 |
| 501 | ENSAPLP00000000740 | zf-C2H2 |
| 502 | ENSAPLP00000010828 | zf-C2H2 |
| 503 | ENSAPLP00000006650 | zf-C2H2 |
| 504 | ENSAPLP00000015701 | zf-C2H2 |
| 505 | ENSAPLP00000015421 | zf-C2H2 |
| 506 | ENSAPLP00000006441 | zf-C2H2 |
| 507 | ENSAPLP00000005131 | zf-C2H2 |
| 508 | ENSAPLP00000013795 | zf-C2H2 |
| 509 | ENSAPLP00000010837 | zf-C2H2 |
| 510 | ENSAPLP00000014469 | zf-C2H2 |
| 511 | ENSAPLP00000014152 | zf-C2H2 |
| 512 | ENSAPLP00000013933 | zf-C2H2 |
| 513 | ENSAPLP00000015705 | zf-C2H2 |
| 514 | ENSAPLP00000015030 | zf-C2H2 |
| 515 | ENSAPLP00000000015 | zf-C2H2 |
| 516 | ENSAPLP00000012437 | zf-C2H2 |
| 517 | ENSAPLP00000001059 | zf-C2H2 |
| 518 | ENSAPLP00000007543 | zf-C2H2 |
| 519 | ENSAPLP00000000696 | zf-C2H2 |
| 520 | ENSAPLP00000001622 | zf-C2H2 |
| 521 | ENSAPLP00000000841 | zf-C2H2 |
| 522 | ENSAPLP00000016198 | zf-C2HC |
| 523 | ENSAPLP00000013899 | zf-C2HC |
| 524 | ENSAPLP00000015776 | zf-GATA |
| 525 | ENSAPLP00000010842 | zf-GATA |
| 526 | ENSAPLP00000004785 | zf-MIZ |
| 527 | ENSAPLP00000014717 | zf-MIZ |

**Table S2. The statistics of annotation with different databases**

| **Database** | **Annotated _Number** | **0<=length**  **<500** | **500<=length**  **<1000** | **1000<=length**  **<1500** | **1500<=length**  **<2000** | **length>**  **2000** |
| --- | --- | --- | --- | --- | --- | --- |
| NT | 88349(98.94%) | 2771 | 7776 | 10122 | 10256 | 57424 |
| Swiss-Prot | 72280(80.95%) | 54960 | 14208 | 2674 | 366 | 72 |
| KOG | 58453(65.46%) | 47075 | 9795 | 1391 | 170 | 22 |
| Total | 219082 | 104806 | 31779 | 14187 | 10792 | 57518 |

KOG: Eukaryotic Orthologous Groups; NT: NCBI Nucleotides. Swiss-Prot:A manually annotated and reviewed protein sequence database

**Table S3. Alignment information for abdominal and subcutaneous fat**

| **Sample** | **Total Reads** | **Mapped reads (ratio)** | **Unique mapped Reads (ratio)** | **Multiple mapped Reads (ratio)** |
| --- | --- | --- | --- | --- |
| Abdomen_A1 | 18441598 | 16700961(85.47%) | 13796287(74.81%) | 1965874(10.66%) |
| Abdomen_A2 | 26595525 | 23885557(84.39%) | 19475841(73.23%) | 2968060(11.16%) |
| Abdomen_A3 | 21813671 | 19601607(85.08%) | 16262510(74.55%) | 2296979(10.53%) |
| Abdomen_A4 | 18498083 | 16657749(85.35%) | 13902507(75.16%) | 1884954(10.19%) |
| Abdomen_A5 | 25522372 | 22847538(84.87%) | 18978002(74.36%) | 2682401(10.51%) |
| Abdomen_A6 | 25279708 | 22492391(83.77%) | 18380967(72.71%) | 2795935(11.06%) |
| Abdomen_B1 | 22232980 | 20601088(89.97%) | 16163317(72.70%) | 3839635(17.27%) |
| Abdomen_B2 | 21496301 | 19597686(88.25%) | 15297021(71.16%) | 3673717(17.09%) |
| Abdomen_B3 | 19934153 | 17771773(85.39%) | 13502099(67.73%) | 3520371(17.66%) |
| Abdomen_B4 | 21393084 | 19044335(85.53%) | 14467932(67.63%) | 3829362(17.90%) |
| Abdomen_B5 | 27154182 | 23939944(83.86%) | 18939319(69.75%) | 3831455(14.11%) |
| Abdomen_B6 | 28737690 | 21623334(85.90%) | 32264(0.11%) | 24654064(85.79%) |
| Abdomen_C1 | 24406941 | 21718150(83.86%) | 17739491(72.68%) | 2728696(11.18%) |
| Abdomen_C2 | 23768869 | 21249107(85.15%) | 17706492(74.49%) | 2533761(10.66%) |
| Abdomen_C3 | 22715479 | 20366899(85.04%) | 16920979(74.49%) | 2396483(10.55%) |
| Abdomen_C4 | 17604122 | 15597433(84.14%) | 12937733(73.49%) | 1874838(10.65%) |
| Abdomen_C5 | 23676096 | 21375004(86.48%) | 18045991(76.22%) | 2429167(10.26%) |
| Abdomen_C6 | 23743681 | 21339156(85.79%) | 17987127(75.76%) | 2381491(10.03%) |
| Abdomen_D1 | 26642071 | 24002098(85.84%) | 20255292(76.03%) | 2613587(9.81%) |
| Abdomen_D2 | 18156305 | 16451994(86.24%) | 13835872(76.20%) | 1822893(10.04%) |
| Abdomen_D3 | 22567111 | 20313068(85.60%) | 16959233(75.15%) | 2358263(10.45%) |
| Abdomen_D4 | 19109965 | 17012031(84.32%) | 14095713(73.76%) | 2018012(10.56%) |
| Abdomen_D5 | 21728652 | 19573667(84.73%) | 15914131(73.24%) | 2496622(11.49%) |
| Abdomen_D6 | 22311331 | 20024731(85.37%) | 16671064(74.72%) | 2376156(10.65%) |
| Abdomen_E1 | 21611699 | 19518356(86.14%) | 16228066(75.09%) | 2388092(11.05%) |
| Abdomen_E2 | 24567312 | 21951048(85.00%) | 18178288(73.99%) | 2704861(11.01%) |
| Abdomen_E3 | 18892887 | 16667697(83.48%) | 13634011(72.16%) | 2138674(11.32%) |
| Abdomen_E4 | 22379235 | 20036198(85.35%) | 16596572(74.16%) | 2504236(11.19%) |
| Abdomen_E5 | 26757639 | 24040063(85.78%) | 19990353(74.71%) | 2962070(11.07%) |
| Abdomen_E6 | 21008046 | 18942826(86.80%) | 16132130(76.79%) | 2102905(10.01%) |
| Abdomen_F1 | 27031805 | 24725920(88.20%) | 18841915(69.70%) | 5000883(18.50%) |
| Abdomen_F2 | 20394322 | 18802165(89.46%) | 14700132(72.08%) | 3544533(17.38%) |
| Abdomen_F3 | 24804720 | 22620803(88.22%) | 17923806(72.26%) | 3958833(15.96%) |
| Abdomen_F4 | 23052337 | 21384200(89.96%) | 17147932(74.39%) | 3589248(15.57%) |
| Abdomen_F5 | 23287514 | 21293872(88.95%) | 16601057(71.29%) | 4112574(17.66%) |
| Abdomen_F6 | 22015150 | 20445316(90.21%) | 16185221(73.52%) | 3674328(16.69%) |
| Subcutaneous_A1 | 26356633 | 21099070(74.96%) | 16180872(61.39%) | 3576595(13.57%) |
| Subcutaneous_A2 | 27143475 | 22077249(76.24%) | 17326009(63.83%) | 3368505(12.41%) |
| Subcutaneous_A3 | 26908431 | 21336005(74.00%) | 16351927(60.77%) | 3559985(13.23%) |
| Subcutaneous_A4 | 27823218 | 22881261(77.10%) | 17859720(64.19%) | 3591977(12.91%) |
| Subcutaneous_A5 | 27757735 | 21908842(73.69%) | 16887924(60.84%) | 3566868(12.85%) |
| Subcutaneous_A6 | 20942227 | 16251079(72.49%) | 12542739(59.89%) | 2638720(12.60%) |
| Subcutaneous_B1 | 23199583 | 19149941(77.78%) | 15121910(65.18%) | 2923147(12.60%) |
| Subcutaneous_B2 | 27991251 | 22340470(75.10%) | 17325131(61.89%) | 3697644(13.21%) |
| Subcutaneous_B3 | 25526154 | 20508046(75.08%) | 15739745(61.66%) | 3425609(13.42%) |
| Subcutaneous_B4 | 27472153 | 22799003(78.10%) | 18005814(65.54%) | 3450502(12.56%) |
| Subcutaneous_B5 | 24107253 | 18087979(69.76%) | 13441577(55.76%) | 3375015(14.00%) |
| Subcutaneous_B6 | 23999350 | 19478160(76.05%) | 15027111(62.61%) | 3225512(13.44%) |
| Subcutaneous_C1 | 25847364 | 21834064(79.46%) | 17381526(67.25%) | 3155963(12.21%) |
| Subcutaneous_C2 | 23530445 | 19717770(78.87%) | 15640792(66.47%) | 2917775(12.40%) |
| Subcutaneous_C3 | 26971031 | 22912623(79.61%) | 18229927(67.59%) | 3241917(12.02%) |
| Subcutaneous_C4 | 31493817 | 26531634(79.55%) | 21339124(67.76%) | 3713121(11.79%) |
| Subcutaneous_C5 | 24668770 | 20942096(79.92%) | 16773468(67.99%) | 2942984(11.93%) |
| Subcutaneous_C6 | 23774367 | 20177328(80.06%) | 16193345(68.11%) | 2841036(11.95%) |
| Subcutaneous_D1 | 28969285 | 24612407(80.16%) | 19692192(67.98%) | 3528458(12.18%) |
| Subcutaneous_D2 | 26724886 | 22569539(79.36%) | 17949926(67.17%) | 3257763(12.19%) |
| Subcutaneous_D3 | 29455602 | 25300305(81.03%) | 20338536(69.05%) | 3528781(11.98%) |
| Subcutaneous_D4 | 26719178 | 22720908(80.14%) | 18142955(67.90%) | 3270427(12.24%) |
| Subcutaneous_D5 | 28222108 | 24276166(81.14%) | 19563321(69.32%) | 3335853(11.82%) |
| Subcutaneous_D6 | 28143827 | 24046760(80.50%) | 19260901(68.44%) | 3394145(12.06%) |
| Subcutaneous_E1 | 26818476 | 21962405(76.89%) | 17123681(63.85%) | 3497129(13.04%) |
| Subcutaneous_E2 | 27904431 | 22969085(77.43%) | 17892882(64.12%) | 3714079(13.31%) |
| Subcutaneous_E3 | 26803331 | 22292769(78.05%) | 17406485(64.94%) | 3513916(13.11%) |
| Subcutaneous_E4 | 27811692 | 23000012(77.81%) | 18052798(64.91%) | 3587708(12.90%) |
| Subcutaneous_E5 | 25133931 | 20709183(77.58%) | 16284089(64.79%) | 3214629(12.79%) |
| Subcutaneous_E6 | 24642459 | 20529850(78.65%) | 16334752(66.29%) | 3045807(12.36%) |
| Subcutaneous_F1 | 25631974 | 21809060(80.22%) | 17448011(68.07%) | 3114284(12.15%) |
| Subcutaneous_F2 | 25898219 | 21650596(78.62%) | 17041193(65.80%) | 3320151(12.82%) |
| Subcutaneous_F3 | 28628664 | 24218034(79.80%) | 19385774(67.71%) | 3461205(12.09%) |
| Subcutaneous_F4 | 29679291 | 24996435(79.50%) | 20114070(67.77%) | 3481380(11.73%) |
| Subcutaneous_F5 | 25597537 | 21610818(79.61%) | 17300358(67.59%) | 3076823(12.02%) |
| Subcutaneous_F6 | 26085359 | 21941686(79.16%) | 17438438(66.85%) | 3211107(12.31%) |
| Mean | 24634946 | 21187006(81.96%) |  |  |

**Table S4. DETs for abdominal and subcutaneous fat**

| **Sample** | **Stage** | **Transcripts counts** | **Up regulated** | **Down regulated** |
| --- | --- | --- | --- | --- |
| Abdominal | -48h vs 0h | 13511 | 8985 | 4526 |
| Abdominal | 0h vs 12h | 10548 | 3487 | 7061 |
| Abdominal | 12h vs 24h | 551 | 252 | 299 |
| Abdominal | 24h vs 48h | 4592 | 2663 | 1929 |
| Abdominal | 48h vs 72h | 15541 | 9952 | 5589 |
| Subcutaneous | -48h vs 0h | 2206 | 1179 | 1027 |
| Subcutaneous | 0h vs 12h | 5811 | 2778 | 3033 |
| Subcutaneous | 12h vs 24h | 1472 | 711 | 761 |
| Subcutaneous | 24h vs 48h | 5639 | 2702 | 2937 |
| Subcutaneous | 48h vs 72h | 4428 | 2368 | 2060 |
| Abdominal vs Subcutaneous | -48h | 14054 | 7068 | 6986 |
| Abdominal vs Subcutaneous | 0h | 12226 | 5006 | 7220 |
| Abdominal vs Subcutaneous | 12h | 11995 | 6433 | 5562 |
| Abdominal vs Subcutaneous | 24h | 12854 | 6800 | 6054 |
| Abdominal vs Subcutaneous | 48h | 12255 | 6240 | 6015 |
| Abdominal vs Subcutaneous | 72h | 18627 | 7853 | 10774 |

**Table S5. List of primers**

| **Transcripts** | **Forward primer (5'‐3')** | **Reverse primer (5'‐3')** |
| --- | --- | --- |
| PPARD-1 | CGTGAAGTCTAGGGCAGTGT | AGCTGTACCCGTCTTCAAATACT |
| PPARD-2 | AGCATCACAGGACAGCTCAGG | GTGCCATTTGAGACGTTCGTTA |
| SMAD3-1 | TCACCATCCCCAGGTCGTTA | AAAGGCGTACTCGCACATCT |
| SMAD3-2 | GTCCTGATGGAGTTTGGTCG | GCGTACTCGCACATCTCCA |
| STAT3-1 | CTTGGGGCCAGGACGG | GTCGCTTCAGAGCCTTTTATTTTG |
| STAT3-2 | CTGACTCGGGTGGTTTCTTTG | GAACTGCATGAGCGAGTCCAG |
| FHL2-1 | CCAGTGCAAGAAGGCTATCAC | CTGATCGGGTTTGTGCATCC |
| FHL2-2 | ATGGGAGTGAGTGGGTGAGT | GAGTAAATGTTGTCCCTGGTGC |
| SLC16A2-1 | CTCCATCTTCGGCATCCACAACTC | AGGAGCCGACCCATGCTGTTT |
| SLC16A2-2 | CACCAGATATTGCTGTGGACCGA | GCCAAGGAGCCGACCCATG |
| GAPDH | AGTCGGAGTCAACGGATTTGG | GCTACCACTTGGACTTTGCC |
